# Spatial spillover impact of determinants on child mortality in Pakistan: evidence from Spatial Durbin Model

**DOI:** 10.1186/s12889-023-16526-6

**Published:** 2023-08-24

**Authors:** Muhammad Ramzan Sheikh, Sami Ullah Khan, Munir Ahmed, Rashid Ahmad, Asad Abbas, Irfan Ullah

**Affiliations:** 1 School of Economics, Bahauddin Zakariya University, Multan, Pakistan; 2 Department of Economics, Gomal University, Dera Ismail Khan, KP Pakistan; 3 Department of Management Sciences, COMSATS University Islamabad, Vehari Campus, Vehari, Pakistan; 4 Department of Economics, COMSATS University Islamabad, Vehari Campus, Vehari, Pakistan; 5 Reading Academy, Nanjing University of Information Science and Technology, Nanjing, China

**Keywords:** Child mortality, Univariate autocorrelation, Spatial clustering, Spatial durbin model, Spatial spillover effect, Pakistan

## Abstract

**Background:**

Child mortality is a major challenge to public health in Pakistan and other developing countries. Reduction of the child mortality rate would improve public health and enhance human well-being and prosperity. This study recognizes the spatial clusters of child mortality across districts of Pakistan and identifies the direct and spatial spillover effects of determinants on the Child Mortality Rate (CMR).

**Method:**

Data of the multiple indicators cluster survey (MICS) conducted by the United Nations International Children’s Emergency Fund (UNICEF) was used to study the CMR. We used spatial univariate autocorrelation to test the spatial dependence between contiguous districts concerning CMR. We also applied the Spatial Durbin Model (SDM) to measure the spatial spillover effects of factors on CMR.

**Results:**

The study results showed 31% significant spatial association across the districts and identified a cluster of hot spots characterized by the high-high CMR in the districts of Punjab province. The empirical analysis of the SDM confirmed that the direct and spatial spillover effect of the poorest wealth quintile and MPI vulnerability on CMR is positive whereas access to postnatal care to the newly born child and improved drinking water has negatively (directly and indirectly) determined the CMR in Pakistan.

**Conclusion:**

The instant results concluded that spatial dependence and significant spatial spillover effects concerning CMR exist across districts. Prioritization of the hot spot districts characterized by higher CMR can significantly reduce the CMR with improvement in financial statuses of households from the poorest quintile and MPI vulnerability as well as improvement in accessibility to postnatal care services and safe drinking water.

**Supplementary Information:**

The online version contains supplementary material available at 10.1186/s12889-023-16526-6.

## Background

The child mortality rate is defined as the risk of a child dying prior to his/her 5^th^ birthday [1]. The eradication of child mortality rate plays a significant role in determining human well-being, prosperity and economic development worldwide, especially in developing countries [2–7]. It is one of the targets (end preventable deaths of new-borns and children under 5 years of age) set by the United Nations Sustainable Development Goals (SDGs), i.e., SDG goal 3; ensure healthy lives and promote well-being for all at all ages [8, 9]. Target 3.2 of SDG goal 3 aims at reducing neonatal mortality to at least 12 deaths per 1000 live births and child mortality to at least 25 deaths per 1000 live births by the year 2030. Governments worldwide are striving to facilitate their people by providing skilled health professionals concerning birth assistance. As a result, the percentage of birth assisted by skilled health professionals has improved from 77 percent in the years 2008–2014 to 84 percent in 2015–2021 [10]. Such progress in the health sector has substantially reduced the global child mortality rate by 14 percent from 2015 to 2020. Despite the reduced global child mortality rate, 5 million children under the age of 5 died before their 5^th^ birthdays in the year 2020 alone [10]. The global child mortality rate has deteriorated by 61 percent, from 93 to 37 deaths per 1,000 live births from 1990 to 2020 [11]. Despite this considerable progress, reducing child mortality remains a significant challenge for health professionals worldwide, particularly in low- and middle-income countries.

Child mortality is a severe public health problem which is determined not only by health-related variables, socioeconomic and demographic characteristics, housing attributes but also influenced by climate change and environmental attributes such as temperature, precipitation, CO_2_ emissions, rainfall, air pollution and droughts [1, 12, 13]. All these attributes vary geographically from one location to another [14, 15]. Temperatures can be very cold in the polar regions and very hot in the equatorial regions. Similarly, rainfall can be plentiful in tropical jungles and scarce in dry deserts [16, 17]. Industrialized countries produce more carbon dioxide than undeveloped countries [18]. Arid and semi-arid regions experience frequent droughts, while coastal and certain tropical regions receive considerable rains [19, 20]. Child mortality rates are affected by all of these factors taken together. Low rainfall and drought can reduce the availability of clean water, which can raise the risk of waterborne diseases, while high temperatures can cause heat-related ailments [21, 22]. Children's respiratory health is negatively impacted by rising CO_2_ emissions, which contribute to air pollution [23]. Droughts and heavy precipitation can also cause agricultural disruption, which in turn can cause food instability, malnutrition, and compromised immune systems, all of which contribute to an increase in infant mortality [24]. Therefore, the necessity for region-specific measures to mitigate these risks and protect vulnerable people is highlighted by the fact that temperature, rainfall, CO_2_ emissions, drought, and precipitation all have an impact on child mortality.

Pakistan being a developing nation, faces several health-related infectious diseases such as diarrhea, malaria, HIV, and pneumonia, which severely contribute to neonatal mortality, infant mortality, and child mortality. Despite the implementation of various health developmental programs in collaboration with the World Health Organization (WHO) and the United Nations International Children's Emergency Fund (UNICEF), such as the "Sehat Sahulat Program" and the "Prime Minister's National Health Insurance Program," child mortality remains prevalent in Pakistan [25–27]. According to information from the World Bank, child mortality in Pakistan has significantly reduced from 140 death per 1000 live births in the year 1990 to 65 deaths per 1000 live birth in 2020 [28]. However, this figure of 65 deaths per 1000 live birth is still far from the United Nation’s SDGs target of 25 deaths per 1000 live births by 2030. Consequently, researchers and health professionals have been compelled to identify the significant determinants of child mortality. In this regard, numerous studies have been conducted to identify the socioeconomic factors that influence the child mortality rate in Pakistan [29–38].

Population growth and rapid urbanization pose a threat to inhabitants’ accessibility to health and health-related social indicators, especially child healthcare facilities [39–42]. In Pakistan, for instance, mothers and babies lack affordable and quality healthcare services, resulting in 75 percent of newborn deaths. These deaths are primarily caused by poor access to immunization and substandard maternal and infant care services [43]. Several socioeconomic characteristics, health and health-related social indicators, housing facilities, environmental attributes, and geographical factors play a significant role in the determining child mortality [1, 12, 13]. Factors such as increasing parental education, household wealth status, access to clean drinking water, toilet facilities and exposure to mass media have been shown to reduce the probability of child mortality [31]. Moreover, child mortality is directly associated with poverty indicators, including income inequality and gross national income [44]. Access to adequate birth spacing between two pregnancies and maternal health care services also significantly reduces the liklihood of infant mortality [45]. However, the management of access to these health and health-related social indicators in Pakistan is inadequate and varies across geographical boundaries [46]. For instance, 50 percent of Pakistan’s population lacks proper access to basic healthcare services, and 42 percent of people do not have health insurance [47]. Additionally, approximately 21 million inhabitants lack access to safe drinking water placing Pakistan among the top ten countries with low access to safe drinking water [48, 49]. Furthermore, only 20 percent of residents have access to liquified petroleum gas (LPG), which is used as a clean fuel for domestic cooking purposes [46]. Inequalities in access to health-related social indicators exist globally, particularly within different geographical localities of a country. These geographical disparities in terms of access to health indicators have a profound impact on the child mortality rate, promoting researchers and health professionals to investigate child mortality from a spatial perspective. This emerging topic has gained considerable attention among health academicians. Therefore, there is a pressing need to identify the impact of various socioeconomic and other health-related social indicators on child mortality from spatial perspectives.

Spatial analysis is mainly based on the first law of geography, as presented by Waldo Tobler in 1970. According to the law, “everything in the world is related to everything else, but distant things are less related than nearer things” [50, 51]. In 1990, the law became the foundation of spatial analysis, which proves beneficial in numerous fields of study, particularly in social and regional sciences [52]. Given the reputation of spatial analysis, understanding the unique localities, characterized by spatial clusters or hotspots, as well as cold spots, and spatial outliers, concerning child mortality, has emerged as a new research topic. These spatial clusters and outliers aid in identifying the regional disparities or inequalities in terms of child mortality. To address and mitigate these regional inequalities, the sustainable development goals (SDGs) outlined in Agenda 2030 call for the reduction of inequality within and among countries, as specified in SDG goal 10 [8, 9]. Therefore, numerous studies have been conducted on the spatial analysis of child mortality [53–62]. These studies utilize various spatial statistical techniques and spatial econometric models to achieve their research objectives and identify geographical disparities related to child mortality. However, in Pakistan, the determinants of child mortality have been poorly investigated, and insufficient attention has been given to assessing the impact of socioeconomic, health, housing, and environmental attributes on child mortality from a spatial perspective. It is crucial to urgently identify the spatial spillover (direct and indirect) effects of these indicators on child mortality.

Bringing it all together, this study represents the first attempt in Pakistan to investigate child mortality using spatial terminology, employing popular spatial techniques such as univariate global and local Moran’s I and the Spatial Durbin Model (SDM). The investigation of spatial clusters and outliers holds great significance for health professionals and the government of Pakistan, as it aids in addressing variations in child mortality across different regions and formulating appropriate policies. This study makes a significant contributes to the existing literature by accomplishing two major objectives. Firstly, it recognizes spatial inequalities in child mortality across districts of Pakistan through the detection of spatial clusters and outliers. Secondly, it measures the spatial spillover effects of various sociodemographic, health, housing, and environmental attributes on child mortality in Pakistan.

## Methodology

### Study area

**Fig. 1:**
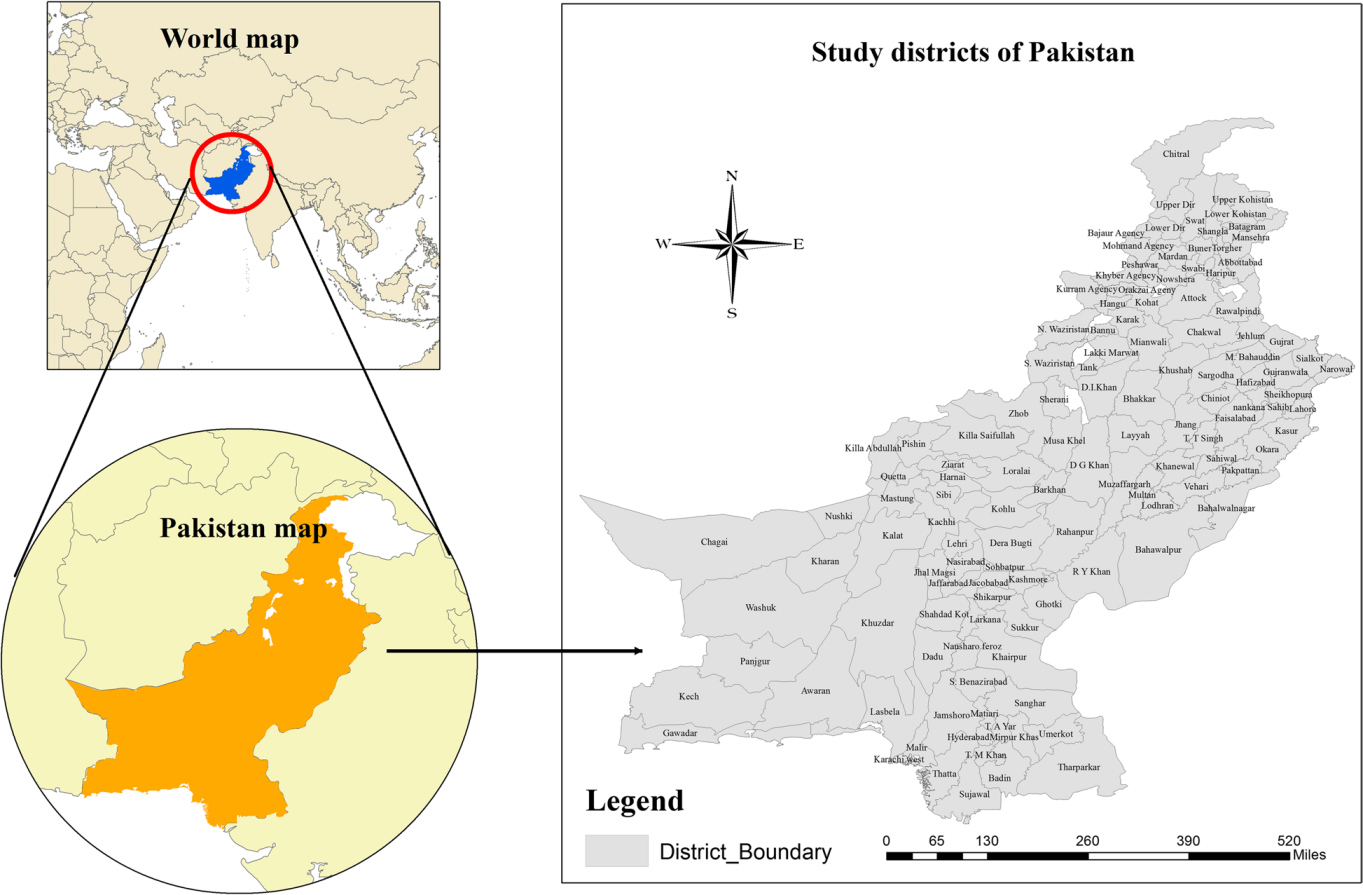
Study area (Study districts of Pakistan)

This study addresses the spatial analysis of child mortality in the districts of Khyber Pakhtunkhwa (KP), newly merged districts of KP previously known as Federally Administered Tribal Areas (FATA), Punjab, Sindh, and Baluchistan provinces of Pakistan. Household level important information on socioeconomic characteristics, housing attributes, health and health-related social information associated with child and mother, environmental indicators, and geographical attributes were extracted from districts of aforementioned provinces of Pakistan. However, some of the districts were omitted from the study either due to the unavailability of data or possessing less information on the study indicators. The remaining districts from all four provinces of Pakistan were considered significant to explore the spatial analysis because previous studies in Pakistan did not investigate the spatial patterns of child mortality. Therefore, these districts were deemed as study observations shown in Fig. 1 as under.

### Data source and sampling design

This study has used the recently conducted Multiple Indicators Cluster Survey (MICS) with the collaboration of the United Nations international children’s emergency fund (UNICEF) in the years 2018 and 2019 for Punjab and KP, FATA, Sindh, and Baluchistan provinces respectively [63, 64]. These surveys contain rich information on various sociodemographic, health, environmental, and housing attributes that can better determine child mortality in Pakistan at the district level. Separate questionnaires for children, women, and household-related information were used to collect the data. A multi-stage, stratified cluster sampling framework was applied to select the survey sample. This frame was based on Pakistan’s population and housing census in the year 2017 [65]. According to this approach, in the first stage, the primary sampling units (PSUs) selected were the enumeration areas (EAs). Households were listed in each EA sample and finally, a sample of 20 households was chosen in the second stage to collect the information from each household.

### Sample size and unit of analysis

MICS adopted multiple stages clustering sampling technique. Although the surveys were conducted at a household level, information at the district level was also gathered. A total of 4,265 EAs were surveyed from the four provinces and information on socioeconomic, demographic, housing, health, environment, etc. were collected from 85,300 households using three separate questionnaires of children, women, and household level. As the present study has mainly focused on child mortality at geographical boundaries, the household-level information concerning the study variables was aggregated at the district level. Therefore, the district was considered as the unit of analysis in the present study. By doing so, 130 districts out of 158 were considered as the sample size.

The unit of analysis in this study is district but information of the study variables has been extracted at household level. Therefore, the household level variables were aggregated at district level by applying the simple mean formula in Eq. (2.1),

2.1 $$Mean= \frac{1}{n}\sum_{i=1}^{n}{VAR}_{i}$$

where n is the number of households i = 1, 2, 3, …, n and ${VAR}_{i}$ is the variable score of i^th^ households.

### Variables of the study

This study mainly focused on measuring the spatial spillover impact of various indicators on child mortality in Pakistan. Therefore, child mortality was considered as the outcome variable, and socioeconomic, health, housing, and environment attributes as explanatory variables of the study. In this regard, a total of 10 (including dependent) indicators were chosen in the present study. The outcome variable was *“child mortality rate at 1000 live birth”* and the explanatory variables were 1) *“poorest quintile”*, 2) *“room density”*, 3) *“mother literacy”*, 4) *“tobacco used by mother”*, 5) *“low weight birth”*, 6) *“postnatal care of newly born children”*, 7) *“access to improved drinking water”*, 8) *“access to improved sanitation facility”*, and 10) *“MPI vulnerability”*. A detailed explanation is provided in Table 1.

**Table 1:** Description of the study variables

S. No	Variable name	Variable abbreviation	Description	Unit	Expected sign
**Dependent variable**					
1	Child mortality	CMR	Number of deaths of children under five years per thousand live births in a district	Numbers	(…)
**Independent variables**					
2	Poorest wealth quintile	PWQ	Percent of households in poorest (20%) quintile according to the wealth index quintile in a district	Percentage	( +)
3	Room density	RD	Average members of households per room in a district	Numbers	( +)
4	Mother literacy	ML	Percent of mothers aged 15–19 years with primary, middle, high or above education in a district	Percentage	(-)
5	Tobacco used by mother	TUM	Percent of mothers aged 15–49 years who uses any tobacco product in a district	Percentage	( +)
6	Low birth weight	LBW	Percent of women aged 15–49 years having live birth with weight lower than 2500gm in a district	Percentage	( +)
7	Postnatal care to newly born child	PNBC	Percent of postnatal health check of newly born children within two days of delivery in a district	Percentage	(-)
8	Access to improved drinking water	IDW	Percent of households with access to improved drinking water facility in a district	Percentage	(-)
9	Access to improved sanitation	IS	Percent of households with access to improved sanitations facility in a district	Percentage	(-)
10	MPI vulnerability	MPIV	Percent of households vulnerable to multidimensional poverty according to MPI in a district	Percentage	( +)

Source: Authors own computations based on Multiple Indicators Cluster Survey (MICS)

### Univariate global and local spatial analysis of child mortality

To test whether the neighboring districts concerning child mortality were spatially associated with one another, global l Moran’s I statistic was used. Moreover, in the presence of spatial associations, where the spatial clusters and/or outliers are located. To assess the location of spatial clusters and outliers, local Moran’s I statistic was used. Statistically, global and local Moran’s I test are written in Eq. (2.2) and Eq. (2.3) respectively as under,

2.2 $$I=\frac{n\sum_{i=1}^{n}\sum_{j=1}^{n}{w}_{ij}({x}_{i}-\overline{x })({x}_{j}-\overline{x })}{\sum_{i=1}^{n}\sum_{j=1}^{n}{w}_{ij}\sum_{i=1}^{n}{(x}_{i}-{\overline{x })}^{2}}, i\ne j$$

2.3 $${I}_{i}=\frac{n({x}_{i}-\overline{x })\sum_{i=1}^{n}{w}_{ij}({x}_{j}-\overline{x })}{\sum_{i=1}^{n}({x}_{i}-{\overline{x })}^{2}}, i\ne j$$

where *n* is the number of districts; *x*_*i*_ and *x*_*j*_ are the child mortality scores of i^th^ and j^th^ districts respectively; $\overline{x }$ represents the mean rate of child mortality of all districts; *w*_*ij*_ is the queen contiguity weight matrix. If i^th^ and j^th^ districts share a common boundary, the assignment *w*_*ij*_ = 1, otherwise *w*_*ij*_ = 0. *I* (in Eq. 1) represent the global univariate Moran’s I score ranges between + 1 and -1, i.e., *1* ≤ *I* ≥ *-1*. When Moran’s I = 0, it means that the child mortality is randomly and/or irregularly dispersed, when Moran’s I > 0, it means that the child mortality is positively agglomerated, and when Moran’s I < 0, it means that the child mortality rates of neighboring regions are negatively associated. To check the significance of Moran statistic, a null hypothesis (H_o_) of spatial randomness was tested against the alternative hypothesis (H_1_) of spatial clusters/patterns.

*I*_*i*_ (in Eq. 2.3) is the extent of spatial association between each i^th^ and its surrounding districts [66, 67] proposed dividing the Local Indicators of Spatial Association (LISA) results into four quadrants, i.e., high-high (H–H) or hot spot, low-low (L-L) or cold spot, high-low (H–L) or outlier, and low–high (L–H) or an outlier. The H–H and L-L quadrants mean that the child mortality rate of a district and its adjacent districts are significantly spatially agglomerated, whereas the quadrants of H–L and L–H mean that the contiguous districts are heterogeneous or randomly dispersed.

### Empirical modeling

Lesage and Pace in 2009 proposed the Spatial Durbin Model (SDM) which includes the lagged dependent variable as well as the independent variables [68]. Generally, the SDM is written in Eq. (2.4) as under,

2.4 $${y}_{i} =\mathrm{\alpha }+\rho W{y}_{i}+\beta {X}_{i}+\theta W{X}_{i}+{\varepsilon }_{i}, {\varepsilon }_{i}\sim N\left(0,{\sigma }_{u}^{2}\right)$$

where i = 1, 2, 3, …, n, ɑ is the constant term, ${y}_{i}$ is a vector of observations on the dependent term, ${{\varvec{X}}}_{{\varvec{i}}}=[{X}_{1i},{X}_{2i},\dots ,{X}_{Ki}]$ is the vector of K independent variables, ${\varvec{\beta}}=[{\beta }_{1},{\beta }_{2},\dots ,{\beta }_{K}]$ is the vector of parameters at explanatory variables, ${\varepsilon }_{i}$ is the error term, $\rho$ is the spatial autoregression parameter, $W$ is the spatial weight matrix of $n\times n$ dimensions with zero diagonal elements, and $\theta$ is the vector of spatial parameters of spatially lagged explanatory variables.

The SDM in Eq. (2.4) is specified to our study variables. The specification of the SDM model in econometric form is written in Eq. (2.5) as under,

2.5 $${CMR}_{i}= \mathrm{\alpha }+\rho \sum_{j=1}^{n}{W}_{ij}{CMR}_{j}+{\beta }_{1}{PWQ}_{i}+{\beta }_{2}{RD}_{i}+{\beta }_{3}{ML}_{i}+{\beta }_{4}{TUM}_{i}+{\beta }_{5}{LBW}_{i}+{\beta }_{6}{PNBC}_{i}+{\beta }_{7}{IDW}_{i}+{\beta }_{8}{IS}_{i}+{\beta }_{9}{MPIV}_{i}+{\theta }_{1}\sum_{j=1}^{n}{W}_{ij}{PWQ}_{j}+{\theta }_{2}\sum_{j=1}^{n}{W}_{ij}{RD}_{j}+{\theta }_{3}\sum_{j=1}^{n}{W}_{ij}{ML}_{j}+{\theta }_{4}\sum_{j=1}^{n}{W}_{ij}{TUM}_{j}+{\theta }_{5}\sum_{j=1}^{n}{W}_{ij}{LBW}_{j}+{\theta }_{6}\sum_{j=1}^{n}{W}_{ij}{PNBC}_{j}+{\theta }_{7}\sum_{j=1}^{n}{W}_{ij}{IDW}_{j}+{\theta }_{8}\sum_{j=1}^{n}{W}_{ij}{IS}_{j}+{\theta }_{9}\sum_{j=1}^{n}{W}_{ij}{MPIV}_{j}+{\varepsilon }_{i} , i\ne j$$

where, ${CMR}_{i}$ is child mortality rate (dependent variable), i and j are the neighboring districts of Pakistan, ${W}_{ij}$ is the spatial weight matrix (symmetrical in the upper right and lower left) explained in Eq. 5 in detail. $\rho$ is the spatially lagged coefficient of child mortality rate reflecting the degree (and direction) of spatial spillover between the contiguous districts. ɑ is the constant term, ${W}_{ij}{CMR}_{j}$ is the spatial autocorrelation matrix of the dependent variable which shows the effect of ${CMR}_{j}$ of j district adjacent to ${CMR}_{i}$ of i district. PWQ, RD, ML, TUM, LBW, PNBC, IDW, IS, and MPIV is respectively the poorest wealth quintile, room density, mother literacy, tobacco used by mother, low birth weight, postnatal care to a newly born child, improved drinking water, improved sanitation, and MPI vulnerability. ${\beta }_{1},{\beta }_{2}, \dots , {\beta }_{9}$ are the coefficients of explanatory variables, ${\theta }_{1}, {\theta }_{2}, \dots , {\theta }_{9}$ are the spatial autocorrelation coefficients of independent variables reflecting the impact of all explanatory variables in the neighbouring districts on the child mortality rate of the local district, and ${\varepsilon }_{i}$ is the random error term normally distributed with zero mean and constant variance.

### Spatial weight matrix

This study uses the spatial queen contiguity weight matrix define as, the districts that share common boundaries as well as a common diagonal. The symmetric spatial weight matrix ${W}_{n\times n}$ for n districts is shown in Eq. (2.6) as under,

2.6 $${W}_{ij}=\left[\begin{array}{ccc}\begin{array}{c}\begin{array}{c}{\omega }_{11}\\ {\omega }_{21}\end{array}\\ \vdots \\ {\omega }_{n1}\end{array}& \begin{array}{c}\begin{array}{c}{\omega }_{12}\\ {\omega }_{22}\end{array}\\ \vdots \\ {\omega }_{n2}\end{array}& \begin{array}{cc}\begin{array}{c}\begin{array}{c}\cdots \\ \cdots \end{array}\\ \vdots \\ \cdots \end{array}& \begin{array}{c}{\omega }_{1n}\\ \begin{array}{c}{\omega }_{2n}\\ \vdots \end{array}\\ {\omega }_{nn}\end{array}\end{array}\end{array}\right]$$

According to the queen contiguity rule, each element of the weighted matrix ${W}_{ij}$ is defined as:

$${W}_{ij}= \left\{\begin{array}{c}1, if\,districs\,i\,and\,j\,are\,the\,neighboring\,districts\\ 0, if\,districts\,i\,and\,j\,do\,not\,share\,a\,common\,boudary\end{array}\right\}$$

## Results

### Visual representation of child mortality

**Fig. 2:**
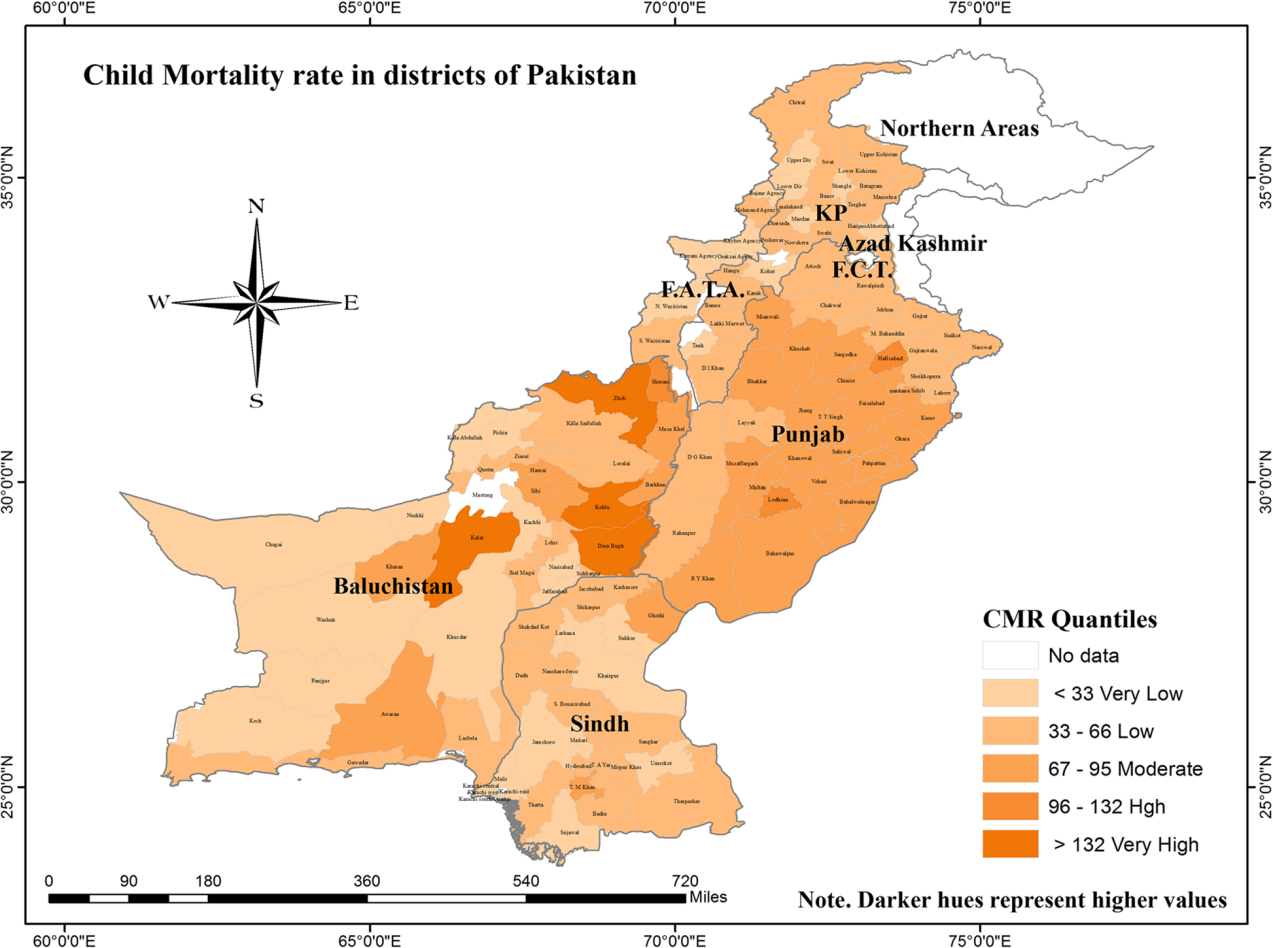
Spatial distribution of child mortality rate in districts of Pakistan. Authors own computation based on Multiple Indicators Cluster Survey (MICS)

Figure 2 depicts the visual representation of CMR in districts throughout four provinces and FATA region of Pakistan. CMR is classified into five quantiles: Very Low, Low, Moderate, High, and Very High. Regions with less than 33 fatalities per 1000 live births were deemed very low quantile districts, whereas areas those with more than 132 deaths per 1000 live births were considerably very high quantile districts. In terms of CMR, almost all districts in Punjab province showed a spatial cluster of low and moderate quantile districts. Similarly, districts in Sindh province presented a mix pattern of very low and low quantile districts. FATA and KP provinces presented slightly varied patterns with a mix of low and moderate quantile districts of Pakistan. In contrast, CMR is highly high in parts of Baluchistan's upper (central) districts while it is very low in the west-southern regions. Both the highest and lowest quantile districts were located in Baluchistan. The highest CMR (of 165 deaths per 1000 live births) was recorded in the Dera Bugti district and the lowest rate was calculated as 10 (deaths per 1000 live births) for district Mastung. Thus, there is extreme heterogeneity in the districts of Baluchistan concerning CMR. Child mortality scores of all districts are presented in additional file, Table A.

### Global and local spatial results of child mortality

**Fig. 3:**
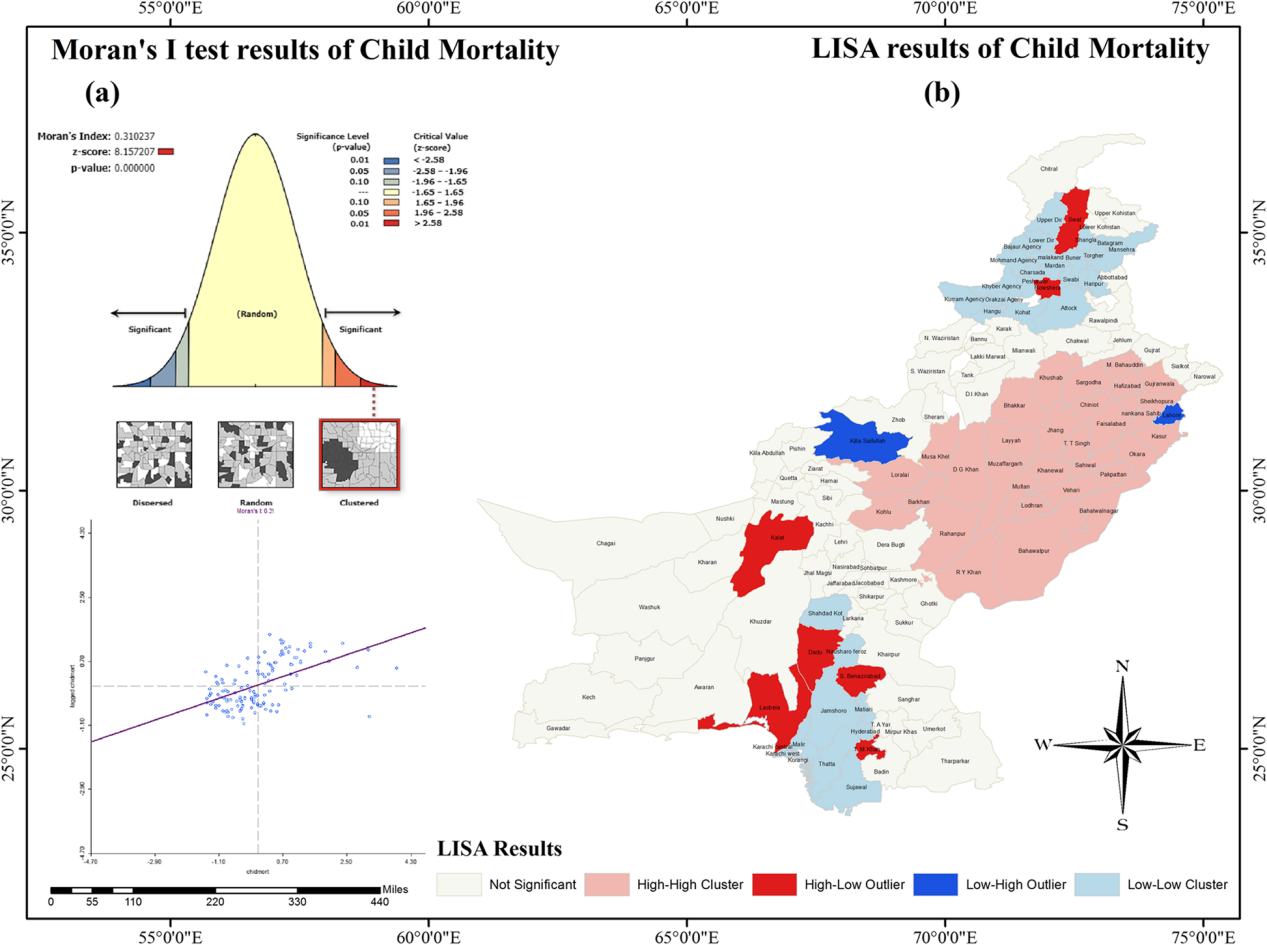
Univariate spatial analysis of (a) Moran's I and (b) LISA tests of child mortality based on Multiple Indicators Cluster Survey (MICS)

As the district is the unit of analysis, the results of both global and local Moran’s I are entirely aggregated at the district level. A combined map was generated to illustrate the spatial associations and spatial clustering of child mortality (Fig. 3). Figure 3a displays the results of global Moran’s I, while Fig. 3b depicts the local results of Moran’s I, also known as Local Indicators of Spatial Association (LISA).

Overall, the global Moran’s Index of spatial autocorrelation of 0.31 (Psuedo *p*-value < 0.01, 999 permutations) indicated a moderate association among the contiguous districts of Pakistan (Fig. 3a). These positive associations are significant with respect to CMR levels in regions with high and/or low focus. In other words, 31 percent of neighbouring districts are spatially clustered (with either a high or low CMR). Therefore, to identify the areas with significant clustering, there is a need to analyze the CMR at the local/district level. From the LISA cluster map (Fig. 3b) it can be seen that there is a significantly bigger clustering of the H–H districts followed by the L-L clustered districts. Moreover, all of the H–H cluster districts were primarily located in Punjab indicating that the high child mortality rate in a specific district of Punjab is significantly surrounded by districts with a high CMR. It is also evident that L-L concentrated districts were mainly located in two distinct loctions. One L-L clustered districts was discovered in the upper districts of KP province, while the other was found in the western and southern districts of Sindh province. These districts are accompanied by districts that have a low CMR. Interestingly, each of the two L-L concentrated districts has a spatially significant H–L outlier, indicating that these districts have a higher CMR but are surrounded by districts with a lower CMR. Finally, one district each in Punjab and Baluchistan provinces was seen with L–H characteristics which confirmed that very few districts have higher CMR surrounded by lower CMR districts.

### Multicollinearity diagnostic test results of study variables

Before attempting to quantify the impact of independent variables on the child mortality rate, it is crucial to test whether the study indicators are multicollinear with one another. The Variance Inflation Factor (VIF) and Tolerance test were applied in this regard (Table 2). As a rule of thumb, any variable is deemed multicollinear if its VIF value exceeds 10 (or its Tolerance value is less than 0.1). Such variables cause issues in regression analysis, resulting in skewed, biased and inefficient outcomes. Our study results revealed that all independent variables had a VIF score < 10 or Tolerance > 0.1, indicating that there is no serious correlation among the explanatory variables of the study.

**Table 2:** Collinearity diagnostics of the study variables

S. No	Variable name	Tolerance	VIF score
1	Poorest wealth quintile	0.332	3.009
2	Room density	0.568	1.760
3	Mother literacy	0.269	3.720
4	Tobacco used by mother	0.724	1.381
5	Low birth weight	0.380	2.629
6	Postnatal care to newly born child	0.669	1.494
7	Access to improved drinking water	0.603	1.657
8	Access to improved sanitation	0.356	2.813
9	MPI vulnerability	0.689	1.451

Dependent variable: Child mortality rate

### Spatial Durbin Model results

With consideration of the spatial spillover impact of explanatory variables on CMR, the conventional Ordinary Least Square (OLS) technique does not produce consistent results, which may lead to inaccurate conclusions. Because simple regression models do not incorporate the average of lag (or neighbouring values of) explanatory variables into account, the size of the influence in direct and indirect effects is not disaggregated. SDM provides the ability to include the lag value of independent variables in the model, allowing the coefficient in direct and indirect (spillover) scores to be separated.

Table 3 shows the spatial spillover results of child mortality. Overall, the model is best fitted as the goodness-of-fit (F-test) is statistically significant at a 1% level of significance. The spatially lagged coefficient ρ is highly significant, demonstrating spatial association between the distribution of CMR in districts of Pakistan, which confirms the rationality of integrating the spatial spillover effects into the econometric model. The poorest wealth quintile and MPI vulnerability have had a favorable impact on CMR, but Postnatal care for newly born children and improved water have had a negative impact. The orientations of the other explanatory variables are consistent, although they do not contribute significantly to the child mortality rate.

**Table 3:** Spatial spillover results of Spatial Durbin Model

Variables	Direct effect		Indirect effect		Total effect	
	**Coefficient**	**z-value**	**Coefficient**	**z-value**	**Coefficient**	**z-value**
Poorest quintile	0.312^b^	2.201	0.571^b^	2.067	0.883^a^	3.219
Room density	1.436	0.359	10.721	1.500	12.157	1.737
Literacy of mother	-0.041	-0.217	-0.611	-1.046	-0.652	-1.043
Tobacco uses by mother	0.108	0.379	0.316	0.695	0.424	0.523
Low weight < 2.5 kg birth	0.088	0.302	0.665	1.189	0.753	1.469
Postnatal care new born	-0.256^b^	-2.020	-1.381^a^	-5.899	-1.637^a^	-6.651
Improved water	-0.401^b^	-2.072	-0.549^b^	-1.984	-0.950^a^	-3.005
Improved sanitation	-0.014	-0.098	-0.207	-0.508	-0.221	-0.591
MPI vulnerable	0.656^b^	2.144	0.047	0.072	0.703^d^	1.952
Constant	40.875	0.769	**–-**	**–-**	**–-**	**–-**
Spatial ρ						0.28^a^
Log-likelihood						-2049.41
AIC						4118.02
BIC						4127.28
Coefficient of determination (R^2^)						0.48
Adjusted R^2^						0.40
F-statistic						5.77^a^

Dependent variable = child mortality rate per 1000 live births

“^a^”,” ^b^”, and”^c^” denote level of significance at “1%”, “5%”, and “10%” respectively

#### The direct and indirect impact of the poorest wealth quintile

The poorest wealth quintile has a positive and considerable direct and indirect effect. On average, a one percent increase in the poorest wealth quintile leads to an increase in the CMR by 0.312 units in a local district, whereas for each incremental percent of the poorest quintile in the neighbouring districts raise the local district’s child mortality rate by 0. 571 units. The total (Direct + Indirect) impact of the poorest quintile is highly significant at a 1% level of significance.

#### *The direct and indirect *impact* of MPI vulnerability*

MPI vulnerability, like the poorest wealth quintile, has positive direct and indirect effects, but the spillover impact of MPI vulnerability is negligible. However, the total impact of MPI vulnerability is significant at a 10% level of significance. On average, an incremental MPI vulnerability in a local district boost child mortality by 0.65 units and a one percent increase in MPI vulnerability in surrounding districts increases CMR by 0.04 units in a local district, although only marginally.

#### *The direct and indirect impact of *postnatal* care to the newly born child*

Unlike the poorest wealth quintile ad MPI vulnerability, the spatial spillover effects of postnatal care on newly born children are inversely associated to the CMR. An additional increase in the percentage of households with postnatal care for newly born children will significantly reduce child mortality by 0.26 units in a local district. Also, each incremental percent of households having access to postnatal care for newly born children in neighboring districts will substantially reduce the CMR by 1.38 units. The total impact of -1.64 is highly significant at a 1% level of significance.

#### The direct and indirect impact of improved water

The spatial impact of improved water availability in households has significantly lowered the child mortality rate in the districts of Pakistan. On average, CMR in a local district is influenced by 0.4 units with a rise in the percentage of households with access to improved water facilities. The indirect effect was also found to be negative which means that on average, a one percent rise in households with access to improved water in neighboring districts will lead to diminishing the CMR by 0.55 units in a local district. Moreover, the total spatial impact of improved water was found to be negative and highly significant at a 1% level of significance.

## Discussion

Child mortality is a matter of significant global importance, particlularly in developing nations such as Pakistan. the eradication of child mortality constitutes a primary objective within the framework of the United Nations SDG 3 [8]. Also, it is the most serious problem in low- and middle-income countries. Concerning CMR, a considerable difference exists between advanced and developing nations. The United Nations endeavors to mitigate such disparities among nations throughout the world as the reduction of inequalities is also a key objective of the 17 SDGs [8]. This study has demonstrated two important outcomes pertaining to the rate of child mortality. The initial findings revealed considerable spatial inequalities, including clusters and outliers, concerning CMR across study districts of Pakistan. Additionally, the study examined the impact of sociodemographic, housing, health, and environmental indicators on child mortality across all districts in Pakistan, specifically focusing on the spatial spillover effects.

There was a notable disparity in the rate of child mortality observed among the districts within the four provinces of Pakistan [69]. The province of Baluchistan exhibited a heterogenous outlier of child mortality where both the highest and lowest CMR districts were located. In contrast, a substantial majority of districts within the Punjab province exhibited a cohesive cluster characterized by comparable levels of child mortality. The observed spatial patterns provide evidence that inequalities in child mortality rates are more pronounced in the province of Baluchistan province when compared to the districts of Punjab province. Similar disparities have been found in previous studies of [69] and [70]. The districts within the province of Baluchistan exhibit a relatively low level of residential access to health and health-related indicators, consequently leading to a higher crude mortality rate (CMR) in these districts [46, 71]. To ascertain the significance of the spatial clusters pertaining to child mortality, it was necessary to conduct global and local autocorrelation analysis. this analysis aimed to determine whether spatial patterns exhibit significant asymmetry or heterogeneity. The study results yielded valuable insights regarding the presence of spatial clusters and/or outliers in terms of CMR. Findings from the study revealed that in terms of child mortality, the contiguous districts of Pakistan were significantly agglomerated by 31 percent. the majority of districts within the Punjab province exhibit a substantially elevated crude mortality rate, with a notable concentration of districts experiencing high rates of child mortality (referred to as the H–H cluster). In comparison to the Punjab region, the districts within the KP province, as well as the western and southern regions of the Sindh province, exhibit a comparatively lower child mortality rate. This phenomenon is notably concentrated in th neighbouring districts, forming a distinct cluster characterized by low CMR (referred to as the L-L cluster). Moreover, it was observed that district with low (high) CMR were predominately located in close proximity to the clusters of districts with high (low) CMR. Punjab province exhibits a relatively higher level of accessibility to health services [46]; however, the spatial analysis conducted in this study reveals a concentration on high mortality rates, following a pattern known as H–H. The potential cause may be partially attributed to the escalating population growth in Punjab [72, 73]; however, the study identified additional factors that predominately influence child mortality [71]. 

Child mortality is widely regarded as a highly sensitive health outcome that serves as a reflection of a country’s healthcare system accessibility and quality, as well as its socio-economic development within the health sector [74]. The findings of the spatial spillover analysis indicate that families belonging to the poorest wealth quintiles have experienced elevated rates of child mortality. The increase in the proportion of the population belonging to the poorest wealth quintile has a positive impact on child mortality rates. This effect is obsereved both within a specific local district (referred to as the direct effect) and across neighbouring districts when considering he average child mortality rates within the poorest wealth quintile (known as spatial spillover effect).. Such positive relationship is also obvious from the previous studies of [75–77]. Pakistan has about 64 percent of rural households [65] and most of these households are categorized in the poorest wealth quintile, which has consequently had a notable impact on child mortality rates. It is evident that about two-third of the inhabitants of Pakistan resides in rural areas, where approximately 40 percent of household experience multidimensionally poverty [78]. The spatial analysis of the study has substantiated that MPI vulnerability has had a direct as well as indirect impact on child mortality in Pakistan. Households that are MPI-vulnerable face a lack of access to multiple essential aspects of daily life, particularly healthcare services [79]. This lack of access ultimately has an impact on the child mortality rate. Our findings indicated a favourale and positive spatial (direct and spillover) impact of MPI vulnerability on CMR in Pakistan. The finding of our study are consistent with prior research on the nexus between poverty and child mortality [80, 81].

The issue of ensuring fair and equal access to healthcare services is a matter of global significance, especially in nations with lower economic resources and middle-income status [82]. Pakistan, as a developing nation, confronts a serious challenge pertaining to the limited availability of health-related services, particularly prenatal and postnatal care to the mothers as well as to the newly born child [46, 83, 84]. The absence of healthcare services, particularly for newly born children, has the potential to increase to a rise in child mortality rates [85, 86]. According to the study outcomes, postnatal care to the newly born child has significantly determined child mortality. The provision of postnatal care to a newly born child in a local district resulted in a proportional decrease in child mortality rates. The spillover effect of postnatal care to a newly born child is also observed to be significant. CMR in a local district is substantially mitigated as access to postnatal care in neighboring districts increases. Previous studies have also substantiated the adverse effects of postnatal care on child mortality in Pakistan [29, 34]. The determination of CMR cannot be solely attributed to access to healthcare services. Environmental attributes, especially the availability of safe drinking water and sanitation, have a major contribution in determining various forms of mortality, maternal, infant, and child mortality [87–89]. Developing countries like Pakistan lack access to safe drinking water. Only 21 percent of inhabitants in Pakistan have access to safe drinking water [49, 90]. The findings of the study have substantiated the notion that the amelioration of and availability of safe drinking water have significantly reduced child mortality within the various districts of Pakistan. The finding also indicated that an expansion in household access to safe drinking water in a local district (direct effect), as well as in neighboring districts (spatial spillover effect), resulted in a decrease in the CMR within that district. Our study results are in line with the previous studies examining the association between child mortality and drinking water [89, 91].

## Limitations

This study encompasses a number of important limitations. First, there may be several other (than the study) indicators at individual, household, and/or community levels to determine child mortality in Pakistan However, this particular study considered only ten indicators based on the data availability. The MICS dataset lacks comprehensive information beyond the study variables, potentially leading to biased and inconsistent estimates. Second, climate-related variables were excluded from the study as the dataset have no information on district level. Third, the statistical analysis was performed at the district level due to the unavailability of household or community-level coordinate information (longitude and latitude) in the dataset. Finally, certain districts were excluded from the study either as a resutld of prevailing law enforcement issues or due to a lack of necessary data to accurately capture the true picture of child mortality.

## Conclusion and recommendations

This paper mainly focused on studying child mortality from spatial perspectives. The researchers conducted an analysis to identify the spatial clusters and outliers of districts in Pakistan with regards to child mortality rate (CMR). Additionally, they measured the spatial spillover impact of significant factors of child mortality. The study’s immediate findings concluded that CMR exhibited a spatial clustering pattern characterized by high-high districts in Punjab province. The aforementioned findings hold significant value for public health practitioners and policy planners, as they provide important insights for determining priorities in addressing spatial inequalities concerning CMR across the districts under study. Based on the empirical findings of the spatial durbin model (SDM), it is also concluded that both the poorest wealth quintile and MPI vulnerability have (directly and indirectly) pumped the child mortality in Pakistan. Given this conclusion, it is imperative to prioritize attention towards families residing in both local and neighboring districts who fall within the poorest wealth quintile and/or exhibit vulnerability according to the MPI. This can be achieved by implementing measures aimed at improving their financial circumstances, with the ultimate goal of reducing the child mortality rate. Moreover, the research findings have substantiated that the provision of enhanced postnatal care to the newly born child and the implementation of improved drinking water systems in both local and surrounding districts have had a significant impact on reducing the CMR in Pakistan. There exists a pressing necessity to improve the accessibility of child (and maternal) healthcare services and safe drinking water in order to mitigate or alleviate child mortality rates in Pakistan.

### Supplementary Information

**Additional file 1:** **Table A.** Child mortality rate of all study districts of Pakistan.

## Data Availability

The datasets of Multiple Indicators Cluster Survey (MICS) are publicly available to all users and researchers. Interested researchers can visit the survey through the given link. https://mics.unicef.org/surveys.

## Abbreviations

SDG: Sustainable Development Goal
UNDP: United Nations Development Programme
UNICEF: United Nations International Children’s Emergency Fund
WHO: World Health Organization
GOP: Government of Pakistan
LPG: Liquified Petroleum Gas
KP: Khyber Pakhtunkhwa
FATA: Federally Administered Tribal Areas
MICS: Multiple Indicators Cluster Survey
SDM: Spatial Durbin Model
MPI: Multidimensional Poverty Index
CMR: Child Mortality Rate
LISA: Local Indicator of Spatial Association
VIF: Variance Inflation Factor
OLS: Ordinary Least Square
