# Supplementary material for: Spatial spillover impact of determinants on child mortality in Pakistan: evidence from Spatial Durbin Model
Source: BMC Public Health. 2023 Aug 24;23:1612. doi: 10.1186/s12889-023-16526-6 (PMC10464234; doi:10.1186/s12889-023-16526-6)
Supplement: Supplementary file 1 — Additional file 1: Table A. Child mortality rate of all study districts of Pakistan. [file 12889_2023_16526_MOESM1_ESM.docx]

**Appendix**

**Table A. Child mortality rate of all study districts of Pakistan**

| **District** | **CMR** | **District** | **CMR** | **District** | **CMR** |
| --- | --- | --- | --- | --- | --- |
| **Federally Administered Tribal Areas (FATA)** | | | | | |
| Bajaur Agency | 24 | N. Waziristan | 11 | S. Waziristan | 56 |
| Kurram Agency | 32 | Orakzai Agency | 26 | Khyber Agency | 31 |
| Mohmand Agency | 39 |  |  |  |  |
| **Baluchistan** | | | | | |
| Awaran | 68 | Kharan | 75 | Panjgur | 14 |
| Barkhan | 73 | Khuzdar | 23 | Pishin | 21 |
| Chagai | 11 | Killa Abdullah | 11 | Quetta | 38 |
| Dera Bugti | 165 | Killa Saifullah | 37 | Sherani | 121 |
| Gawadar | 44 | Kohlu | 142 | Sibi | 79 |
| Harnai | 84 | Lasbela | 64 | Washuk | 19 |
| Jaffarabad | 22 | Loralai | 53 | Zhob | 133 |
| Jhal Magsi | 50 | Mastung | 10 | Ziarat | 46 |
| Kachhi | 18 | Musa Khel | 73 | Lehri | 58 |
| Kalat | 143 | Nasirabad | 27 | Sohbatpur | 52 |
| Kech | 27 | Nushki | 24 |  |  |
| **Khyber Pakhtunkhwa (KP)** | | | | | |
| Abbottabad | 25 | Karak | 39 | Shangla | 13 |
| Bannu | 62 | Kohat | 58 | Swabi | 52 |
| Buner | 52 | Upper Kohistan | 50 | Swat | 57 |
| Batagram | 49 | Lakki Marwat | 18 | Tank | 14 |
| Charsada | 39 | Lower Dir | 40 | Torgher | 35 |
| Chitral | 47 | Malakand | 50 | Upper Dir | 27 |
| D.I.Khan | 34 | Mansehra | 28 | Peshawar | 38 |
| Hangu | 40 | Mardan | 57 | Lower Kohistan | 58 |
| Haripur | 38 | Nowshera |  |  |  |
| **Punjab** | | | | | |
| Attock | 49 | Jehlum | 61 | Okara | 84 |
| Bahalwalnagar | 71 | Kasur | 80 | Pakpattan | 72 |
| Bahawalpur | 72 | Khushab | 87 | R Y Khan | 67 |
| Bhakkar | 79 | Lahore | 44 | Rahanpur | 62 |
| Chakwal | 43 | Layyah | 61 | Rawalpindi | 42 |
| Chiniot | 95 | Lodhran | 107 | Sahiwal | 77 |
| D G Khan | 64 | M. Bahauddin | 66 | Sargodha | 78 |
| Faisalabad | 72 | Mianwali | 80 | Sheikhopura | 64 |
| Gujranwala | 53 | Multan | 71 | Sialkot | 61 |
| Gujrat | 58 | Muzaffargarh | 87 | T. T Singh | 75 |
| Hafizabad | 110 | nankana Sahib | 82 | Vehari | 85 |
| Jhang | 90 | Narowal | 53 | Khanewal | 95 |
| **Sindh** | | | | | |
| Badin | 47 | Mirpur Khas | 42 | Sujawal | 20 |
| Dadu | 63 | Nausharo Feroz | 36 | Umerkot | 26 |
| Ghotki | 74 | Sanghar | 55 | Karachi central | 28 |
| Hyderabad | 39 | Shahdad Kot | 40 | Karachi west | 16 |
| Jacobabad | 49 | S. Benazirabad | 59 | Karachi south | 41 |
| Jamshoro | 20 | Shikarpur | 54 | Malir | 28 |
| Kashmore | 63 | Sukkur | 33 | Korangi | 18 |
| Khairpur | 27 | T. A Yar | 34 | Karachi east | 27 |
| Larkana | 13 | T. M Khan | 69 | Thatta | 47 |
| Matiari | 30 | Tharparkar | 52 |  |  |
